
JOURNAL INFORMATION
==============================
NLM Title Abbreviation: J High Energy Phys
Journal ID: J High Energy Phys
NLM Journal ID: 101668727

Journal of High Energy Physics

ISSN: 1126-6708
EISSN: 1029-8479

ARTICLE INFORMATION
==============================
PMCID: PMC12748303
PMID: 41476912
DOI: 10.1007/JHEP12(2025)149
Article ID: 27851
Article version: 1
Subjects: Erratum

Erratum to: The landscape of complexity measures in 2D gravity

http://orcid.org/0000-0003-0533-1223 Cáceres Elena 1
http://orcid.org/0009-0002-4414-5773 Carrasco Rafael rafael.carrasco@ift.csic.es
1 2
http://orcid.org/0000-0002-7254-5427 Patil Vaishnavi 1
http://orcid.org/0000-0002-1426-642X Pedraza Juan F. 2
http://orcid.org/0000-0001-6108-6045 Svesko Andrew 3
1 https://ror.org/00hj54h04 grid.89336.37 0000 0004 1936 9924 Theory Group, Weinberg Institute, Department of Physics, University of Texas at Austin, 2515 Speedway, Austin, Texas 78712 USA
2 https://ror.org/022r8mj40 grid.501798.2 0000 0004 0561 6576 Instituto de Física Teórica UAM/CSIC, Calle Nicolás Cabrera 13–15, 28049 Madrid, Spain
3 https://ror.org/0220mzb33 grid.13097.3c 0000 0001 2322 6764 Department of Mathematics, King’s College London, Strand, London, WC2R 2LS U.K.
Electronic publication date: 2025 Dec 18
Print publication date: 2025
Volume: 2025
Issue: 12
Electronic Location ID: 149
Received 2025 Nov 21; Accepted 2025 Nov 21
Copyright: © The Author(s) 2025
Copyright year: 2025
License: Open Access. This article is distributed under the terms of the Creative Commons Attribution License (CC-BY 4.0), which permits any use, distribution and reproduction in any medium, provided the original author(s) and source are credited.
License URL: https://creativecommons.org/licenses/by/4.0/

Erratum for: 10.1007/JHEP10(2025)218

Funding: https://doi.org/10.13039/100000001 National Science Foundation (NSF) PHY-2210562 Cáceres Elena

Funding: https://doi.org/10.13039/100012818 Comunidad de Madrid 2020-T1/TIC-20495 Cáceres Elena

Funding: Spanish Research Agency FPU22/01262 Cáceres Elena

Funding: Spanish Research Agency CEX2020-001007-S Cáceres Elena

Funding: Spanish Research Agency PID2021-123017NB-I00 Cáceres Elena

Funding: CSIC iMOVE mobility program fellowship 24101 Cáceres Elena

Funding: https://doi.org/10.13039/501100000271 Science and Technology Facilities Council ST/X000753/1 Cáceres Elena
issue-copyright-statement: © SISSA, Trieste, Italy 2021

==============================
Data Availability Statement. This article has no associated data or the data will not be deposited.

Code Availability Statement. This article has no associated code or the code will not be deposited.

Erratum to: JHEP10(2025)218

ArXiv ePrint: 2503.20943

The online version of the original article can be found at 10.1007/JHEP10(2025)218

Publisher’s Note

Springer Nature remains neutral with regard to jurisdictional claims in published maps and institutional affiliations.

